# Evolution of SARS-CoV-2-Neutralizing Antibodies after Two Standard Dose Vaccinations, Risk Factors for Non-Response and Effect of a Third Dose Booster Vaccination in Non-Responders on Hemodialysis: A Prospective Multi-Centre Cohort Study

**DOI:** 10.3390/jcm10215113

**Published:** 2021-10-30

**Authors:** Frank-Peter Tillmann, Lars Figiel, Johannes Ricken, Hermann Still, Christoph Korte, Grete Plassmann, Philipp von Landenberg

**Affiliations:** 1 Medical Centre Cologne-Merheim, Department of Medicine I—Nephrology, Transplantation & Medical Intensive Care, University Witten/Herdecke, D-51109 Cologne, Germany; 2 Centre for Nephrology and Hypertensiology Ibbenbüren, Gravenhorsterstr. 1, D-49477 Ibbenbüren, Germany; still@dialysen-muensterland.de; 3 Centre for Nephrology and Hypertensiology Emsdetten, D-48282 Emsdetten, Germany; figiel@dialysen-muensterland.de (L.F.); korte@dialysen-muensterland.de (C.K.); 4 Centre for Nephrology and Hypertensiology Rheine, D-48431 Rheine, Germany; ricken@dialysen-muensterland.de (J.R.); plassmann@dialysen-muensterland.de (G.P.); 5 LADR GmbH MVZ Nord-West, D-48465 Schüttorf, Germany; p.landenberg@ladr.de

**Keywords:** SARS-CoV-2, COVID-19, vaccination, neutralizing antibodies, antibody titer, hemodialysis, risk factors for non-response, booster vaccination

## Abstract

The aim of this investigation was to determine the effect of SARS-Cov-2 vaccination in hemodialysis patients, search for risk factors for non- or low-response, and to measure the effect of a third booster vaccination in non- or low-responders. Methods SARS-CoV-2 IgG antibodies and the virus-neutralizing capacity were measured 4–5 weeks after a full standard vaccination in 95 chronic hemodialysis patients and 60 controls. IgG titers > 30 AU/mL served to classify participants as responders. Multivariable binary logistic regression analysis was used to search for risk factors of reduced vaccination success. Patients with vaccination failure were offered a third booster dosage. Results 82.1% of the patient cohort as compared to 98.3% of the healthy control group were able to mount SARS-CoV-2 titers above 30 AU/mL after two standard vaccine doses. Mean IgG antibody titers were lower in hemodialysis patients than controls (78 ± 35 vs. 90 ± 20 AU/mL, *p* = 0.002). Multivariable binary logistic regression analysis showed age and immunosuppressive medication as major risk factors for vaccination failure with a decreased probability of successful vaccination of −6.1% (95% CI −1.2 to −10.9) per increase in age of one year and −87.4% (95% CI −98.0 to −21.5) in patients on immunosuppressive therapy (crude odds ratio for vaccination failure for immunosuppressive therapy 6.4). Ten out of 17 patients with non-response to vaccination were offered a third dose. Booster vaccination after the second dose induced an increase in effective antibody titers of >30 AU/mL in seven out of ten patients 4–5 weeks later (70%). Conclusion Standard SARS-CoV-2 vaccination schemes are highly effective in mounting protective neutralizing IgG antibodies in chronic hemodialysis patients. Nevertheless, response to vaccination is diminished as compared to a healthy control group. Major risk factors for vaccination failure are older age and immunosuppressive therapy. In non- or low-responders to standard vaccination a third booster vaccination was able to induce effective antibody titers in about 70% of patients, indicating that a third booster vaccination might be preferable to decreasing immunosuppressive therapy.

## 1. Introduction

In December 2019, the first case of pneumonia caused by a novel zoonotic virus was published [1]. The rapid pandemic spread of this virus, combined with early reports on high mortality rates in special risk groups, has led to an unprecedented global effort to rapidly develop and deploy a safe and effective vaccine. By the end of 2020, a total of 51 clinical SARS-CoV-2 vaccination trials had been registered worldwide [2]. In the production process of these vaccines, manufacturers made use of numerous different principles such as classical inactivated wild viruses, but also next-generation methods such as nucleoside-modified mRNA principles or non-replicating adenoviruses-vectored methods [2]. Other difficulties were to rapidly develop valid diagnostic tools for antibody screening and to search for effective treatment options in severely compromised patients [3].

In the context of this global pandemic, numerous high-risk groups for severe clinical course after SARS-CoV-2 infection have been identified so far. Among these, end-stage renal disease patients (ESRD) on thrice weekly hemodialysis have been identified as an especially vulnerable patient population due to reduced possibility of self-isolation and the well-known impaired immune competence. Correspondingly high mortality rates between 16 and 37% have already been reported in this population from various countries and healthcare systems [4,5]. Although seroconversion rates in hemodialysis patients after two doses of SARS-CoV-2 vaccination have been reported well above the success rates of other conventional vaccination schemes e.g., for hepatitis B in this patient population, these response rates are still below those of the normal population [6,7,8]. The situation is further complicated by the fact that low-symptom infection courses have been reported in dialysis patients despite successful vaccination [9,10]. Furthermore, the questions as to which risk factors lead to a reduced response to vaccination in dialysis patients and whether a third booster-vaccination could lead to a successful formation of antibodies remain still unanswered. Therefore, this prospective multicenter cohort study was performed to investigate the response rates to standard SARS-CoV-2 vaccination schedules, search for risk factors for non-response, and evaluate the effectiveness of a third booster vaccination in non- or low-responders in a cohort of 95 chronic hemodialysis patients.

## 2. Methods

This is a prospective, interventional, multi-center cohort investigation in 95 hemodialysis patients >18 years of age. The control group comprised of 60 healthy staff members of the dialysis units. Patients were recruited in three hemodialysis centers in Germany. Patient classified to participate in this study if they were vaccinated with two doses of either mRNA SARS-CoV-2 vaccine (BNT162b2, Pfizer-BioNTech, Mainz, Germany) or replication-defective viral vector carrying pathogen gene (ChAdOx1 nCoV-19, Oxford-AstraZeneca, Cambridge, UK) at least 3 weeks prior to study inclusion. Anti-SARS-CoV-2 antibodies were re-evaluated 4–5 weeks after the second or after the third vaccination cycle in selected patients. Patients with prior SARS-CoV-2 infection were not eligible to participate in this study. Post-vaccination analysis included the measurement of SARS-CoV-2 IgG-antibody titers and an evaluation of the neutralizing capacity of the IgG-antibody as described below. Patients had been vaccinated either in central vaccination facilities, by their primary care physicians or the dialysis facility itself. Dates of vaccination, type of vaccination used, and person-related data were stored centrally in a password-protected data sheet. Patients classified as non- or low-responders were offered a third booster-vaccination with BNT162b2 after detailed information and written consent.

Past medical history of COVID-19 and outcomes before the start of the study were determined by the medical staff of the facility in all participants prior to the start of the study. Demographic data (age, sex, dialysis vintage, prior history of transplantation, online conductivity Kt/V clearance [OCM-device™ Fa. Fresenius Medical Care], estimated glomerular filtration rate according to the CKD-EPI formula in mL/min/1.73 m^2^ BSA, albumin in mg/L, type of hemodialysis access [fistula, graft, catheter], type of dialysis membrane, candidacy for renal transplantation, active immunosuppressive medication at the time of antibody titer evaluation, diabetes mellitus, and active malignancy) were recorded in every patient at baseline. Major outcome variables were IgG-antibody titers categorized in AU/mL and their neutralizing capacity in percent.

The study was approved by the local ethics committee “Ethikkommission der Ärztekammer Westfalen-Lippe und der Westfälischen Wilhelms Universität” in Münster (2021-131-f-S) and conducted in line with the Declaration of Helsinki and the European Union Clinical Trials Directive 2001/20/EC (EU CTD). Written informed consent to participate and to publish was obtained from all individual participants included in the study. All patients gave informed consent prior to study participation and before a third booster vaccination.

### 2.1. Laboratory Measurements of SARS-CoV-2 Antibodies

#### 2.1.1. SARS-CoV-2 IgG Antibody Test Assay

In this study, a commercially available immunoassay was used for antibody detection, the anti-SARS-CoV-2 S-RBD IgG (Snibe Diagnostics, New Industries Biomedical Engineering Co., Ltd. Snibe, Shenzhen, China). SARS-CoV-2 S-RBD IgG is a chemiluminescent immunoassay (CLIA) that determines IgG Ab against the RBD of the Spike (S) protein of the virus, in human serum or plasma. All analyses were performed on MAGLUMI™ 4000 instrument (Snibe Diagnostics, Shenzhen, China), with results expressed in AU/L (multiplication of antibody titers in AU/mL by 4.3 yields equivalent results in BAU/mL). The assay has a clinical sensitivity between 74.5% (days post onset of Symptoms 0–7) and 100% (days post onset of Symptoms >15), and a specificity of 99.6% (95% confidence interval [95% CI] 98.7%–100%). Results were reported in AU/L from 0 to 100. Values greater than 100 were reported <100 AU/L. For analysis, these data were categorized into three classes of IgG-levels of 0, 1–100, and >100 AU/L.

#### 2.1.2. SARS-CoV-2 IgG-Neutralizing Test Assay

We used the ELISA-based GenScript SARS-CoV-2 Surrogate Virus Neutralization Test Kit (GenScript 105 Biotech, Piscataway Township, NJ, USA). The test was used according to the manufacturer’s recommendations. Samples were diluted in sample buffer and incubated at 37° for 30 min in the 96-well microtiter plates provided, followed by the respective wash and incubation cycles, including controls, and required reagents. The microtiter plates are coated with the “host cell receptor” angiotensin-converting enzyme 2 (ACE2). Samples containing SARS-CoV-2-neutralizing antibodies block the protein-protein reaction between ACE2 and the added (S)-RBD-horseradish peroxidase conjugate. The reduced color change upon addition of chromogenic substrate can be measured photometrically. Optical density (OD) was measured at 450 nm using the microplate reader of a VIRCLIA^®^ automation system. The signal to cut-off ratio was calculated and the values printed and interpreted according to the manufacturer’s protocol and results were reported in %. *Definition of neutralizing capacity of SARS-CoV-2 IgG levels after the second dose*: A value of ≥30% about six weeks after the second vaccination cycle was considered “fully neutralizing capacity” as opposed to “missing or incomplete neutralizing capacity”.

*Definition of responder, low-responder, and non-responder status after the second vaccination dose*: A value of ≥30 AU/mL about four to five weeks after the second and third vaccination cycle was considered “seroconversion or responder status”, 1–30 AU/mL was considered “low-responder status”, and 0 AU/mL “non-responder status”.

### 2.2. Statistical Analyses

Data are shown as mean plus minus standard deviation (SD) or percentage, according to the type of variable analyzed. We used the Chi-Squared test for associations between qualitative variables and a t-test, ANOVA, or the Mann–Whitney-U test for quantitative variables. Multivariable binary logistic regression analysis was performed to search for risk factors for a combined outcome parameter of non- and low-response status after vaccination. Values of *p* < 0.05 were considered statistically significant. Statistical analyses were performed using SPSS, IBM Corp., Armonk, NY, USA.

## 3. Results

### 3.1. Patient and Control Group Characteristics

In total 95 hemodialysis patients were enrolled in this investigation. About 60 staff members served as control group. The major group characteristics of both groups are shown in Table 1. As the control group was composed mainly of staff members it differed substantially from the patient cohort in age, sex, and comorbidities. The patient group was mainly vaccinated using two doses of BNT162b2 (96.8%), whereas the control group was vaccinated with different schemes of BNT162b2 (11.7%), ChAdOx1 nCoV-19 (40.0%), or a combination of both (48.3%). The difference in time between the first and second vaccination dosage was mainly driven by the fact that the staff members started their vaccination primarily with a vector-based vaccine, whereas almost all patients were immunized using a mRNA-based type of vaccine (96.8%).

### 3.2. SARS-CoV-2 IgG Antibody Titers and Neutralizing Capacity after the Second Dose

**Figure 1:**
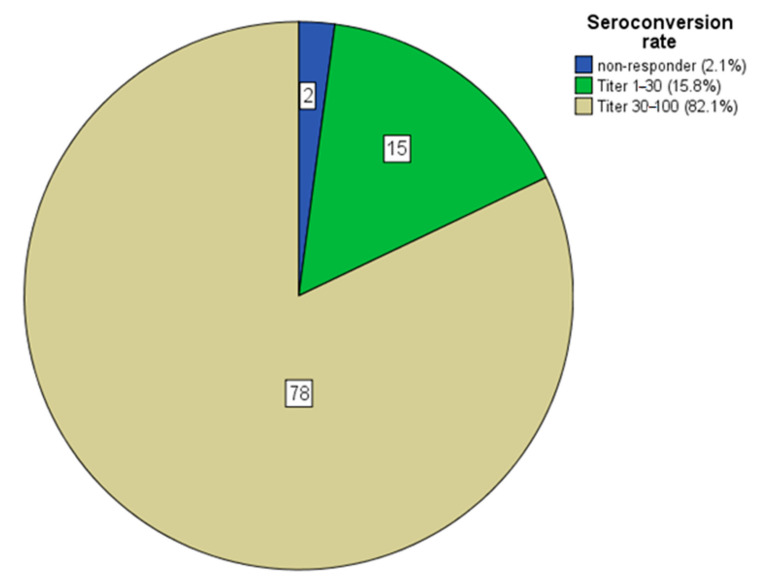
Seroconversion rates in numbers and percent in categories in 95 ESRD patients on hemodialysis after two doses of SARS-CoV-2 vaccination, in the control group only one person (1.7%) showed a low titer between 1 and 30 AU/mL. ESRD = end-stage-renal-disease.

About 4–5 weeks after application of the second dose 2 patients were classified as non-responder (2.1%), 15 patients as low-responder (15.8%), and 78 as responder (82.1%) (Figure 1). In the control group the respective data were 0 (0%), 1 (1.7%), and 59 (98.3%, *p* = 0.009). The healthier control group was also able to mount a higher antibody titer response (90 ± 20 vs. 78 ± 35 AU/mL, *p* = 0.002) than the hemodialysis cohort.

### 3.3. Risk Factor Analysis for Non- or Low-Response to Standard Vaccination

About 17 patients showed reduced antibody titers after SARS-CoV-2 vaccination. Both groups differed significantly with respect to the following variables: Kt/V *p* = 0.011, albumin *p* = 0.009, age *p* = 0.014, and HD-vintage *p* = 0.0017 (Table 2). Univariate binary logistic regression analysis pointed toward lower albumin levels (*p* = 0.003), age (*p* = 0.042), and immunosuppressive therapy (*p* = 0.047) as potential risk factors for non-response. On multivariable binary logistic regression (Table 3) analysis age and immunosuppressive therapy remained strong predictors for vaccination failure with a reduced probability for vaccination success of −6.1% (95% CI −1.2 to 10.9) per increase in age of 1 year and −87.4% (95% CI −98.0 to −21.5), crude odds ratio of 6.4 for immunosuppressive medication (Hosmer-Lemeshow test *p* = 0.504).

### 3.4. Characteristics of Non- and Low-Responders after the Second Dose and after the Third (Booster) Dose

In sum, 17 patients (76.5% males) were classified as non- or low-responders. Nevertheless, only two patients showed no detectable antibody response at all after a full vaccination. Responders were on average younger, had higher albumin levels, were longer on chronic hemodialysis, and showed higher Kt/V values than non- and low-responder (Table 2). In total ten out of 17 non- and low-responders agreed to receive a third (booster) vaccination and the respective characteristics are shown in Table 4. Antibody titer measurement was performed on average 29.1 days after the booster dosage and revealed reassuring antibody titers (six patients > 100 AU/mL, 1 patient between 30–100 AU/mL, and two patients were not able to mount a titer even after the third dosage). Of note both patients who did not mount an antibody response were on immunosuppressive therapy.

### 3.5. Comparison of Vaccination Effectiveness in the Control Group

Because of potential side effects of e.g., cerebral vein thrombosis in young women after vaccination with ChAdOx1 nCoV-19, staff members of the control group were switched to a hybrid vaccination protocol using the mRNA vaccine BNT162b2 as second dosage (48.2%). Comparison between the three vaccination schemes showed lower results in control members who were vaccinated with two doses of ChAdOx1 nCoV-19, whereas vaccination with two doses of BNT162b2 or a hybrid scheme showed similar effectiveness (Table 5).

## 4. Discussion

This study aimed to:

(i) Investigate the effectiveness of standard two-dose vaccination against SARS-CoV-2,
(ii) To search for risk factors of vaccination failure, and,
(iii) To evaluate the effect of a third booster vaccination in hemodialysis patients unable to mount protective antibody titers after standard dose vaccination.

So far, observational reports on SARS-CoV-2 vaccination in chronic hemodialysis patients have shown both, an insufficient immune response [11,12] as well as similar effectiveness when compared to the general population with reported effectiveness rates of >90% after a second dose [13]. Although the response rate of 82.1% in our multi-center cohort was significantly lower than in the healthy control group (98.3%), SARS-CoV-2 vaccination is by far more effective than the so far reported different other vaccinations e.g., for hepatitis B in this vulnerable patient cohort [14]. Furthermore, we feel that when seroconversion rates of hemodialysis patients are compared to mostly healthy staff members as in this and prior studies, it should be kept in mind that healthy staff personnel do not represent an ideal control group. Instead, it would be more sensible to compare the data to a control group with similar age and disease burden. Nevertheless, given the reduced effectiveness of the vector-based vaccine ChAdOx1 nCoV-19 in members of the control group, the difference in antibody titers between the patient and the control group might have been even larger.

Our data are like reported seroconversion rates of 85% [15] or even 91% [16]. Furthermore, we could confirm the neutralizing nature of the measured antibody. Recently, a large cohort study from Germany not only found a seroconversion rate of >95% in hemodialysis but also a comparably high T-cellular immunity [17]. Like our investigation, this study found that immunosuppressive medication represents a major risk factor for non-response to vaccination with an odds-ratio of about 10. Our analysis differs from this large cohort investigation in several ways. First, we could not find a significant effect of dialysis vintage on seroconversion rates, instead our data pointed toward a relevant effect of age. We assume that these differences are mainly driven by the large difference in patient numbers, but nevertheless would suggest further research to finally clarify these points. 

In patients who were not able to mount effectively high antibody titers both, reducing potentially immunosuppressive drugs or applying a so far still off-label use of a third booster vaccination have been discussed in the literature. As most immunosuppressive drugs were given to patients in our cohort to preserve residual renal function after kidney transplant failure, we decided to offer a third booster vaccination. Reassuringly, this procedure resulted in effective antibody titers in seven out of ten non-responders. Furthermore, despite a thorough search for potential side effects the booster dose was well tolerated without any minor or major side effects in all ten patients. Data on the effect of booster vaccinations in hemodialysis patients have yet been scarce. One analysis of 45 hemodialysis patients showed a response rate of 89% after application of two standard doses of BNT162b2. Among the five non-responders, two patients mounted robust antibody titers after a third booster vaccination using the same vaccine brand [18]. Therefore, it seems sensible to suggest booster vaccination over reducing immunosuppressive medications in non-responders on chronic hemodialysis. Out of 17 non-responders in our cohort, 11 were men. We attribute this to the relatively small number of cases but consider it useful to investigate this gender discrepancy in further clinical trials.

## 5. Conclusions

In conclusion, we found high seroconversion rates after standard two doses of SARS-Cov-2 vaccination in chronic hemodialysis patients. Detected antibodies showed a high neutralizing capacity. Concomitant immunosuppressive medication was a major risk factor for vaccination failure, whereas the effects of age and dialysis vintage might be subject to further research. A third booster vaccination in non-responders proved to be highly effective with a response rate of about 70% without detectable side effects. Potential gender differences in response rates to SARS-CoV-2 vaccination might be a subject of further research.

## Figures and Tables

**Table 1:** Basic cohort characteristics. HD = hemodialysis, Kt/V = measured by online-conductivity measurement device OCM™ Fa. Fresenius Medical Care, KTx = kidney transplantation, PMMA = polymethylmetacrylate membrane, IS = immunosuppressive, eGFR = CKD-EPI formula in mL/min/1.73 m^2^ BSA. Continuous variables are shown as mean ± standard deviation, categorical variables are shown in numbers and percent. Statistical analyses were performed by t-test for unpaired variables for continuous data and Chi-square test for categorical variables.

Cohort Characteristics and Response Rates to SARS-CoV-2 Vaccination			
Variable	HD Group (*n* = 95)	Control Group (*n* = 60)	*p*-Value
Low-responder < 30 (AU/mL) (%)	17.9	1.7	0.002
CoV-IgG titer (AU/mL)	78 ± 35	92 ± 20	0.002
Neutralizing capacity (%)	<30 (8.4) 30–75 (19.0) 75–100 (72.6)	<30 (0.0) 30–75 (10.0) 75–100 (90.0)	0.015
Kt/V	1.32 ± 0.39	/	/
eGFR	7 ± 4	94 ± 16	<0.001
Albumin (mg/L)	3609 ± 474	4429 ± 415	<0.001
Age (years)	66.5 ± 15.1	49.2 ± 12.6	<0.001
Time 1 to 2 vaccination (days)	40 ± 9	79 ± 16	<0.001
Time from 2 vaccination to CoV-IgG titer (days)	31 ± 5	31 ± 6	0.863
HD-vintage (years)	4.7 ± 3.9	/	/
Male sex (%)	66.3	8.3	<0.001
HD-filter (%)	polysulfon (90.5) PMMA (9.5)	/	/
Prior KTx (%)	11.6	0.0	<0.001
KTx waitlisted (%)	36.8	/	/
IS-therapy (%)	13.7	0.0	0.003
Diabetes (%)	29.5	1.7	<0.001
Active malignancy (%)	4.2	1.7	0.383
Vaccine (%)	mRNA (96.8) vector (2.1) vector/mRNA (1.1)	mRNA (11.7) vector (40.0) vector/mRNA (48.3)	<0.001
HD-access (%)	shunt (63.2) PTFE (9.5) catheter (27.4)	/	/

**Table 2:** Comparison between responders and reduced-responders defined as CoV-IgG-titers <30 AU/mL in 95 ESRD patients on hemodialysis. HD = hemodialysis, Kt/V = measured by online-conductivity measurement device OCM™ Fa. Fresenius Medical Care, KTx = kidney transplantation, PMMA = polymethylmetacrylate membrane, IS = immunosuppressive, eGFR = CKD-EPI formula in ml/min/1.73 m^2^ BSA. Continuous variables are shown as mean ± standard deviation; categorical variables are shown as percent. Statistical analyses were performed by Mann–Whitney-U test for unpaired variables for continuous data and Chi-square test for categorical variables. n.a. = test statistics not applicable as some cells within the contingency table have expected counts of <5.

Risk Factors for Vaccination Non-Response in 95 ESRD Patients			
Variable	Responder (*n* = 78)	Non- and Low-Responder (*n* = 17)	*p*-Value
CoV-IgG titer (AU/mL)	92 ± 19	14 ± 11	0.000
Neutralizing capacity (%)	<30 (1.3) 30–75 (10.2) 75–100 (88.5)	<30 (41.2) 30–75 (58.8) 75–100 (0.0)	0.000
Kt/V	1.37 ± 0.39	1.12 ± 0.31	0.011
eGFR	7 ± 4	8 ± 4	0.200
Albumin (mg/L)	3646 ± 489	3438 ± 359	0.009
Age (years)	65.0 ± 14.8	73.4 ± 15.1	0.014
Time 1 to 2 vaccination (days)	41 ± 8	35 ± 10	0.041
Time from 2 vaccination to CoV-IgG titer (days)	31 ± 4	32 ± 6	0.706
HD-vintage (years)	4.9 ± 4.1	2.4 ± 1.9	0.017
Male sex (%)	64.1	76.5	0.328
HD-filter (%)	polysulfon (91.0) PMMA (9.0)	polysulfon (88.2) PMMA (11.8)	0.722
Prior KTx (%)	10.3	17.5	0.388
KTx waitlisted (%)	39.7	23.5	0.209
IS-therapy (%)	10.3	29.4	0.037
Diabetes (%)	30.8	23.5	0.553
Active malignancy (%)	0.0	23.5	n.a.
Vaccine (%)	mRNA (97.4) vector (2.6) vector/mRNA (0.0)	mRNA (94.1) vector (0.0) vector/mRNA (5.9)	n.a.
HD-access (%)	shunt (68.0) PTFE (9.0) catheter (23.0)	shunt (41.2) PTFE (11.8) catheter (47.0)	n.a.

**Table 3:** On univariable logistic regression analysis albumin (RC-B = −0.046, *p* = 0.042, Exponent-B = 0.955, 95% CI 0.913–0.998) age (RC-B = 0.002, *p* = 0.003, Exponent-B = 1.002, 95% CI 1.001–1.003) and IS-therapy (RC-B = −1.294, *p* = 0.047, Exponent-B = 0.274, 95% CI 0.077–0.981) were shown to be potential risk factors for vaccination non-response. On multivariable binary logistic regression analysis age and immunosuppressive therapy remained strong predictors for vaccination failure with a reduced probability for vaccination success of −7.3% per increase in age of 1 year and −90.3% for immunosuppressive medication. RC-B = regression coefficient, 95%-CI = 95% confidence interval of exponent-B. KTx = kidney transplantation, IS = immunosuppressive, eGFR = CKD-EPI formula in ml/min/1.73 m^2^ BSA, n.a. = not applicable.

Risk Factors for Vaccination Non-Response in 95 ESRD Patients					
Variable	RC-B	*p*-Level	Exponent-B	95%-CI	
Sex	0.701	0.346	2.015	0.469	8.668
IS-therapy	−2.071	0.026	0.126	0.020	0.785
Kt/V	1.152	0.334	3.164	0.306	32.766
Albumin (mg/L)	0.001	0.664	1.000	0.999	1.002
Age (years)	−0.063	0.016	0.939	0.891	0.988
HD-vintage (years)	0.092	0.439	1.097	0.868	1.386

**Table 4:** Out of 17 individuals with reduced response to standard SARS-CoV-2 vaccination ten patients agreed to receive a third booster vaccination. No local or systemic side effects or signs of myocarditis were noticed after application of the third dosage. Only two patients on immunosuppressive therapy did not mount neutralizing antibodies. SARS-CoV-2 titers in AU/mL, NTC = neutralizing capacity of mounted antibodies in percent, days-2 and days-3 = time from the second and third (booster) vaccination to antibody measurement, ST = steroids, tac = tacrolimus, cyc = cyclophosphamide.

No.	Sex	Vaccine	Age (y)	Titer-2	NTC-2	Days-2	Titer-3	NTC-3	Days-3	IS-Therapy
1	Female	mRNA	49.6	1.6	<30	39	0.0	<30	31	ST/tac
2	Male	mRNA	68.1	4.3	<30	27	13.0	54	30	-
3	Male	mRNA	87.4	28.3	47	43	>100	100	27	-
4	Male	mRNA	61.5	0.0	<30	28	>100	82	36	ST/cyc
5	Male	mRNA	87.5	0.0	<30	25	1.6	<30	27	ST
6	Male	mRNA	79.2	11.4	<30	25	69.1	77	27	-
7	Male	mRNA	84.3	3.7	40	39	>100	96	27	-
8	Male	mRNA	87.2	28.9	38	31	>100	99	30	-
9	Male	mRNA	83.8	12.0	<30	33	>100	93	27	-
10	Male	mRNA	81.7	17.7	47	31	>100	100	29	-

**Table 5:** Comparison between different vaccination schemes in participants of the control group (Scheme 0) and antibody titers (ANOVA *p* = 0.021) after vector-based vaccination and comparable results after mRNA-based and/or hybrid vaccination. Vaccination schemes: 11.7% BNT162b2, 40.0% ChAdOx1 nCoV-19, and 48.3% hybrid.

Response to SARS-CoV-2 Vaccination in the Control Group (*n* = 60)			
Variable	CoV-IgG Titer (AU/mL)	Neutralizing Capacity (%)	Low-Responder <30 (AU/mL) (%)
BNT162b2	99 ± 8	<30 (0.0) 30–75 (0.0) 75–100 (100.0)	0.0
ChAdOx1 nCoV-19	83 ± 27	<30 (0.0) 30–75 (25.0) 75–100 (75.0)	4.2
ChAdOx1 nCoV-19/BNT162b2	97 ± 12	<30 (0.0) 30–75 (00.0) 75–100 (100.0)	0.0

## Data Availability

Data might be available on relevant request.
